
JOURNAL INFORMATION
==============================
NLM Title Abbreviation: J Orthop Traumatol
Journal ID: J Orthop Traumatol
NLM Journal ID: 101090931

Journal of Orthopaedics and Traumatology : Official Journal of the Italian Society of Orthopaedics and Traumatology

ISSN: 1590-9921
EISSN: 1590-9999
Publisher: Springer

ARTICLE INFORMATION
==============================
PMCID: PMC4417836
DOI: 10.1007/s10195-012-0186-y
Article ID: 186
Article version: 1
Subjects: Acknowledgement to Referees (Addendum)

2011 Scientific Referees

Electronic publication date: 2012 Feb 18
Print publication date: 2012 Mar
Volume: 13
Issue: 1
First page: 55
Last page: 55
Copyright: © The Author(s) 2012
License: This article is distributed under the terms of the Creative Commons Attribution License which permits any use, distribution, and reproduction in any medium, provided the original author(s) and the source are credited.
License URL: https://creativecommons.org/licenses/by/4.0/

issue-copyright-statement: © The Author(s) 2012

==============================
Open Access

This article is distributed under the terms of the Creative Commons Attribution License which permits any use, distribution, and reproduction in any medium, provided the original author(s) and the source are credited.

